# Intrasession and Intersession Reliability of Flexibility Tests During Developmental Years: The Effects of Sport, Age, and Sex

**DOI:** 10.3390/sports13080238

**Published:** 2025-07-22

**Authors:** Nikolaos Tsiakaras, Konstantina Karatrantou, Christos Batatolis, Konstantinos Papavasileiou, Fenia Tzeli, Vassilis Gerodimos

**Affiliations:** Department of Physical Education and Sports Science, University of Thessaly, 42100 Trikala, Greece; ntsiakar@uth.gr (N.T.); kokaratr@uth.gr (K.K.); batatoli@uth.gr (C.B.); kostaspapavasiliou10@gmail.com (K.P.); fanitzeli1@gmail.com (F.T.)

**Keywords:** range of motion, reproducibility, test–retest, measurement, sit and reach, back scratch

## Abstract

Flexibility plays an important role in both daily life and performance in various sports. This study evaluated the intrasession and the intersession reliability of flexibility measurements, examining the effects of sport, age, and sex. The sample included 80 wrestling athletes (40 boys: 20 children/20 adolescents; 40 girls: 20 children/20 adolescents), 80 taekwondo athletes (40 boys: 20 children/20 adolescents; 40 girls: 20 children/20 adolescents), and 80 non-athletes (40 boys: 20 children/20 adolescents; 40 girls: 20 children/20 adolescents). The participants performed two assessment sessions, which included two tests (back scratch/sit and reach). The results showed high intrasession and intersession reliability for boys and girls among wrestling and taekwondo athletes (children: ICC = 0.988–0.998, SEM% = 2.31–7.44; adolescents: ICC = 0.993–0.999, SEM% = 1.13–5.19). Additionally, the results demonstrated good/high intrasession and intersession reliability for boys and girls among non-athletes (children: ICC = 0.992–0.997, SEM% = 3.40–9.98; adolescents: ICC = 0.996–0.998, SEM% = 2.81–8.94). The SEM% values were slightly higher in non-athletes vs. athletes (wrestling, taekwondo), as well as in children vs. adolescents, indicating that athletes and adolescents present better reliability than non-athletes and children, respectively. No differences in reliability were observed between boys and girls. In conclusion, the sit and reach and the back scratch are reliable tests in assessing flexibility during the developmental ages. It seems that age and engagement in sports affect the reliability of measurements.

## 1. Introduction

Flexibility is one of the most important components of physical fitness and plays an important role in both athletic performance and everyday life [1,2,3]. An important factor that influences flexibility is participation in different sports and physical activities [1,3]. Wrestling [4,5] and taekwondo [6,7] are two sports where flexibility plays a significant role in the effective execution of various skills that are important for performance enhancement. However, due to their intense nature, both sports show a high incidence of injury, especially at the knee and/or shoulder joints [8,9,10,11,12,13]. Furthermore, low levels of flexibility, often combined with reduced lengths of the hamstring muscles, which are frequently observed in athletes of combat sports and martial arts (e.g., wrestling and taekwondo), can cause various musculoskeletal problems, such as low back pain [14], dysfunction of the sacroiliac joint [15], hamstring injuries [16], patellofemoral pain syndrome [17], and patellar tendinopathy [18]. Additionally, the upper limbs (especially the shoulders) constitute a region of the body that also shows a significant frequency of injuries in both wrestling and taekwondo sports (with injury rates up to 25%) [12,13]. Taking all the above into account, the reliable evaluation of lower- and upper-body flexibility may be used for physical fitness monitoring and training planning, as well as for injury prevention, in young wrestling and taekwondo athletes.

In the scientific literature, there are several tests (field or laboratory) that are used to evaluate flexibility in different muscle groups of the lower and upper body [19,20]. In the present study, we selected the sit and reach test and the back scratch test (also called the zipper test or shoulder stretch test), which are widely used in developmental years (with easily accessible indicative values/norms in different age groups), convenient, simple in execution for various populations, and cost-effective (with portable equipment). Although the above tests are widely used in the scientific literature to assess flexibility in children and/or adolescent non-athletes [21,22,23,24,25,26], as well as athletes of different sports [6,7,27,28,29,30,31,32], there is limited information regarding their intrasession (among trials on the same day) and intersession (between two different days, i.e., test–retest) reliability, especially in young athletic populations. In more detail, previous studies have examined the reliability of the sit and reach test in untrained children and/or adolescents, reporting moderate to high reliability [24,25], while, in athletes, this information is more limited [27,28]. Regarding the reliability of the back scratch test, there is adequate information in healthy or illness-affected older populations [33,34,35,36], whereas, in youths, there is limited information about its reliability [26,28]. Specifically, one study in adolescent tennis players [28] and one study in untrained children and adolescents [26] showed high reliability (ICC = 0.88–0.99) for the shoulder stretch test.

To the best of our knowledge, no previous study has examined and compared the reliability of flexibility tests between young wrestlers, taekwondo athletes, and non-athletes, investigating simultaneously the effects of the sport activity, age, and sex. The reliability, however, of measurements could be influenced by different factors, such as the training status, physical fitness level, age, etc. [37,38,39,40]. Differences in mood, motivation, learning effects, ability to focus on the task, and performance levels, as well as biomechanical factors, may account for these differences in reliability [38,39]. For example, a previous study [41] in young soccer players (11–19 years old) showed different Cronbach’s alpha values among age groups, ranging from 0.72 for the sub-11 age group to 0.94 for the sub-13 age group and 0.93 for the sub-18 age group, during the modified Thomas flexibility test. It could be hypothesized that the neural maturation from childhood to adolescence (leading to changes in the ability of individuals to focus on the task) [42] could impact the overall performance level, as well as the reliability of measurements of neuromuscular performance parameters. Regarding the effect of sex on reliability, one previous study [43] that was performed in adolescents (boys and girls) and used the back-saver sit and reach test demonstrated a borderline significant sex difference in systematic bias, where girls showed slightly greater systematic bias than boys.

Other factors that could influence the reliability of measurements are the number of testing trials performed (one trial, three trials, etc.) and the different methods (average or best value) used to determine the results of measurements. Although, in flexibility tests, there is inadequate information regarding the reliability of measurements using different numbers of trials, in other physical fitness parameters (e.g., handgrip strength), there is a debate on this topic [39,44,45]. Some previous studies have reported that the mean of three trials has higher test–retest reliability than either a single trial or the best of three trials in healthy individuals [39,44], as well as in individuals with intellectual disabilities [45]. At the same time, other studies have observed that a single trial and the mean or the best of two or three trials are equally reliable in determining maximal handgrip strength [46,47,48].

Therefore, the main objective of this study was to evaluate the intrasession and intersession reliability (using various absolute and relative reliability indices) of flexibility measurements (sit and reach test, back scratch test) during the developmental years, examining and comparing the effects of the sport activity (wrestling, taekwondo, no participation in organized physical activity), age (children, adolescents), and sex (boys, girls) on the reliability values. We also examined whether the number of testing trials and the method used for the determination of performance in flexibility tests (single trial, best of three trials, or average of three trials) affected their reliability. We hypothesized that the two flexibility tests (sit and reach test back scratch test) would yield reliable measurements during the developmental years. We also hypothesized that athletes would present slightly better reliability than non-athletes, and adolescents would present slightly better reliability than children. Finally, we hypothesized that a single trial would present slightly lower test–retest reliability than the best and the average of three testing trials.

## 2. Materials and Methods

### 2.1. Participants

**Figure 1:**
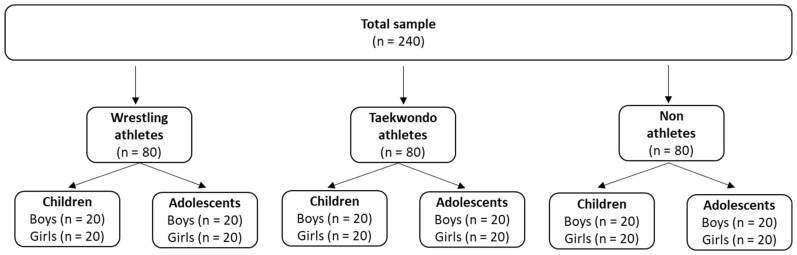
Sample of the study.

Two hundred and forty (240) children (8–10 years old) and adolescents (13–15 years old) voluntarily took part in the current research, where 80 were wrestling athletes, 80 were taekwondo athletes, and 80 were non-athletes (Figure 1). The ages and the anthropometric and training characteristics of the participants (per sport and age group) are presented in Table 1. Wrestling and taekwondo athletes had at least one year of training experience in wrestling or taekwondo and a training frequency of at least 3 times per week, while non-athletes did not systematically engage in any form of exercise. All participants were healthy, with no injuries in the upper and lower limbs for at least 6 months before the commencement of the study. The children’s and adolescents’ parents were informed about the experimental procedures and signed an informed consent form. The current research was conducted according to the Declaration of Helsinki and approved by the Ethics Committee of the University of Thessaly.

### 2.2. Measures

#### 2.2.1. Anthropometric Characteristics

Body mass was assessed using a calibrated physician’s scale (Seca model 755, Seca, Hamburg, Germany), and body height was assessed using a telescopic height rod (Seca model 220, Seca, Hamburg, Germany), according to the recommendations of the American College of Sports Medicine [20].

#### 2.2.2. Flexibility Tests

Flexibility was assessed using two widely used and recognized tests in the developmental years: (a) the sit and reach test and (b) the back scratch test (Table 2).

### 2.3. Design and Procedures

All measurements were performed in the Training and Physical Conditioning Lab of the Department of Physical Education and Sport Sciences of the University of Thessaly by the same investigator. The main investigator of the present research was a physical education teacher and scientific team member of the Training and Physical Conditioning Lab of the DPESS of the University of Thessaly, with extensive experience in the testing and evaluation of physical performance (flexibility, strength, coordination abilities, aerobic capacity). However, it should be mentioned that, before the start of the study, the investigator performed numerous preliminary pilot measurements to familiarize themselves with the two flexibility tests used in the present study (sit and reach test, back scratch test). Furthermore, since the environmental conditions can significantly affect the results of flexibility tests, to ensure reliable and accurate test results, we maintained stable environmental conditions during all measurements (adequate lighting, temperature of 23–25 °C, humidity of 50–55%, proper ventilation, low noise levels), according to the guidelines of the American College of Sports Medicine [20]. The participants visited the laboratory three times. During the first visit, the participants were informed of and familiarized with the flexibility tests. The investigator gave standardized verbal instructions regarding the technique of each test and a visual demonstration of each test using printed photos (such as those presented in Table 2) to all participants.

Furthermore, during the first visit, basic anthropometric characteristics (body height and body mass) were assessed and the hand preference was determined. During the second and third visits, the participants performed the flexibility tests (sit and reach test, back scratch test). Both test and retest sessions were performed at the same time of the day (10:00 a.m.–12:00 p.m.) to prevent potential confounding effects of daily biorhythms. There is evidence that the time interval between test and retest sessions could affect the reliability [19]. To assess test–retest reliability in physical fitness, a time interval of 2 to 7 days is generally recommended by several investigators. This time interval balances the potential for learning/recall or fatigue effects (i.e., the first test may influence the second if the interval is too short) and the possibility of genuine changes in flexibility (if the interval is too long). Taking all the above into consideration, we used a time interval of 72 h between the test and retest. Participants were asked to follow their normal diets for two days before the study, to abstain from intense exercise activity for 24 h before the study, and to have sufficient rest the night before the study. During both the test and retest sessions, before the initiation of the main testing protocol, the participants performed a standardized 5 min warm-up protocol. During the warm-up protocol, the participants performed a 1 min static run of moderate intensity at around 60–65% of the age-predicted maximum heart rate, which was controlled using a heart rate monitor (Polar Electro, Kempele, Finland); 2 min of static and dynamic stretching of the muscles involved in the flexibility tests; and 2 preliminary submaximal trials for each test.

### 2.4. Statistical Analysis

The IBM SPSS Statistics v.28 software (IBM Corporation, Armonk, NY, USA) was used to analyze the data. Before the start of the study, we estimated, using the formula of Walter et al. [49], that a sample size of 20 participants per age group would be adequate for this study (power: 80%; minimum acceptable reliability ICC: 0.7; expected reliability ICC: 0.90). In the present study, the intrasession reliability (reliability among trials on the same day) and intersession reliability (reliability between the first and second testing occasions—test–retest—using different methods for the determination of flexibility, such as a single trial or the mean or the best of three testing trials) were examined, using various relative (intraclass correlation coefficient (ICC) with 95% CI) and absolute (standard error of measurement (SEM), SEM%, 95% limit of agreement (95% LOA)) reliability indices. The standard error of measurement (SEM) was calculated using the following equation: SEM = SD × (1 − ICC), where SD = the sample standard deviation and ICC = the calculated intraclass correlation coefficient [50]. The SEM was divided by the mean of the two measurements and multiplied by 100 to give a percentage value (SEM%) [39]. The LOA was calculated using the following equation: LOA = intertrial mean difference ± 1.96 SD of the intertrial difference [50]. For the ICC values, (i) <0.5 indicated poor reliability, (ii) between 0.5 and 0.75 indicated moderate reliability, (iii) between 0.75 and 0.90 indicated good reliability, and (iv) above 0.9 indicated high reliability [51]. Additionally, for the SEM values, (i) <5% indicated high reliability, (ii) >5% and <10% denoted good reliability, (iii) equal to 10% indicated moderate reliability, and (iv) >10% indicated low reliability.

A repeated-measures analysis of variance (one-way ANOVA) was used to evaluate possible differences in the flexibility tests among the three testing trials on the same day. Paired *t*-tests were used to determine possible differences in flexibility measurements between tests and retests. A three-way ANOVA was used to examine the effects of sport, age, and sex on anthropometric and training characteristics. Sidak’s multiple comparisons were used to locate significantly different means. The level of significance was set at *p* < 0.05.

## 3. Results

### 3.1. Intrasession Reliability

#### 3.1.1. Wrestling Athletes

##### Boys

The repeated-measures analyses of variance showed non-significant differences among the three testing trials for the sit and reach test and back scratch test in children (*p* = 0.14–0.36) and adolescents (*p* = 0.22–0.59). The systematic bias ranged from −0.58 to +0.03 cm in children and from −0.30 to +0.15 cm in adolescent boys. The relative and absolute reliability among trials was high for children in the sit and reach test (ICC = 0.997; SEM% = 2.42) and back scratch test (ICC = 0.998; SEM% = 4.62–5.66). Furthermore, adolescents showed high relative reliability and absolute reliability in the sit and reach test (ICC = 0.998; SEM% = 1.67) and back scratch test (ICC = 0.998; SEM% = 2.57–4.10). Regarding the comparison among age groups, children demonstrated higher SEM% values (2.42–5.66%) than adolescents (1.67–4.10%) in both tests (with greater differences observed in the back scratch test), indicating that adolescents presented better reliability than children. The performance in the sit and reach and back scratch tests per trial (mean ± standard deviation), as well as the relative (ICC with 95% CI) and absolute (SEM, SEM%) reliability indices, are presented in Table 3.

##### Girls

The repeated-measures analyses of variance showed non-significant differences among the three trials for the sit and reach test and back scratch test in children (*p* = 0.15–0.94) and adolescent girls among wrestling athletes (*p* = 0.13–0.40). The systematic bias ranged from −0.14 to +0.25 cm in children and from −0.20 to +0.71 cm in adolescents. The relative and absolute reliability among trials was high for children in the sit and reach test (ICC = 0.988; SEM% = 2.71) and back scratch test (ICC = 0.990–0.993; SEM% = 4.66–6.73). Furthermore, adolescent girls showed high relative reliability and absolute reliability in the sit and reach test (ICC = 0.995; SEM% = 1.90) and back scratch test (ICC = 0.993–0.995; SEM% = 3.56–4.60). Concerning the comparison among age groups, children demonstrated higher SEM% values (2.71–6.73%) than adolescent girls (1.90–4.60%) in both tests (with greater differences observed in the back scratch test), indicating that adolescents presented better reliability than children. The performance in the flexibility tests per trial (mean ± standard deviation), as well as the relative (ICC with 95% CI) and absolute (SEM, SEM%) reliability indices, are presented in Table 4.

#### 3.1.2. Taekwondo Athletes

##### Boys

The repeated-measures analyses of variance showed non-significant differences among the three testing trials for the sit and reach test and back scratch test in children (*p* = 0.20–0.58) and adolescent (*p* = 0.13–0.25) taekwondo athletes. The systematic bias ranged from −0.67 to +0.33 cm in children and from −0.38 to +0.37 cm in adolescent boys. The relative and absolute reliability among trials was high for children in the sit and reach test (ICC = 0.998; SEM% = 2.31) and back scratch test (ICC = 0.996–0.998; SEM% = 5.90–6.91). Additionally, adolescents showed high relative reliability and absolute reliability in the sit and reach test (ICC = 0.998; SEM% = 1.99) and back scratch test (ICC = 0.998; SEM% = 3.77–4.98). Regarding the comparison among age groups, children demonstrated higher SEM% values (2.31–6.91%) than adolescents (1.99–4.98%) in both tests (with greater differences observed in the back scratch test), indicating that adolescents presented slightly better reliability than children. The performance in the sit and reach and back scratch tests per trial (mean ± standard deviation), as well as the relative (ICC with 95% CI) and absolute (SEM, SEM%) reliability indices, for boy taekwondo athletes are presented in Table 5.

##### Girls

The repeated-measures analyses of variance showed non-significant differences among the three trials in the sit and reach test and back scratch test in children (*p* = 0.11–0.54) and adolescent girl taekwondo athletes (*p* = 0.12–0.49). The systematic bias ranged from −0.14 to 0.20 cm in children and from −0.30 to +0.24 cm in adolescents. The relative and absolute reliability among trials was high for children in the sit and reach test (ICC = 0.996; SEM% = 1.77) and back scratch test (ICC = 0.997; SEM% = 4.99–7.44). Moreover, adolescent girls showed high relative reliability and absolute reliability in the sit and reach test (ICC = 0.998; SEM% = 1.13) and back scratch test (ICC = 0.996–0.998; SEM% = 3.82–5.19). Concerning the comparison among age groups, children demonstrated higher SEM% values (1.77–7.44%) than adolescent girls (1.13–5.19%) in both tests (with greater differences observed in the back scratch test), indicating that adolescents presented better reliability than children. The performance in the flexibility tests per trial (mean ± standard deviation), as well as the relative (ICC with 95% CI) and absolute (SEM, SEM%) reliability indices, in girl taekwondo athletes are presented in Table 6.

#### 3.1.3. Non-Athletes

##### Boys

The repeated-measures analyses of variance showed non-significant differences among the three testing trials in the sit and reach test and back scratch test in children (*p* = 0.12–0.18) and adolescent (*p* = 0.14–0.20) non-athletes. The systematic bias ranged from −0.45 to +0.42 cm in children and from −0.36 to +0.27 cm in adolescent boys. The relative and absolute reliability among trials was good to high for children in the sit and reach test (ICC = 0.994; SEM% = 4.55) and back scratch test (ICC = 0.992–0.997; SEM% = 8.94–9.76). Furthermore, adolescents showed good to high relative reliability and absolute reliability in the sit and reach test (ICC = 0.997; SEM% = 2.92) and back scratch test (ICC = 0.996–0.998; SEM% = 4.73–8.94). Regarding the comparison among age groups, children demonstrated higher SEM% values (4.55–9.76%) than adolescents (2.92–8.94%) in both tests, indicating that adolescents presented slightly better reliability than children. The performance in the sit and reach and back scratch tests per trial (mean ± standard deviation), as well as the relative (ICC with 95% CI) and absolute (SEM, SEM%) reliability indices, for boy non-athletes are presented in Table 7.

##### Girls

The repeated-measures analyses of variance showed non-significant differences among the three testing trials in the sit and reach test and back scratch test in children (*p* = 0.10–0.35) and adolescent (*p* = 0.15–0.40) non-athletes. The systematic bias ranged from −0.24 to +0.20 cm in children and from −0.25 to +0.24 cm in adolescent girls. The relative and absolute reliability among trials was good to high for children in the sit and reach test (ICC = 0.996; SEM% = 3.41) and back scratch test (ICC = 0.998; SEM% = 9.79–9.98). Moreover, adolescents showed good to high relative reliability and absolute reliability in the sit and reach test (ICC = 0.997; SEM% = 2.81) and back scratch test (ICC = 0.997–0.998; SEM% = 7.18–9.08). Regarding the comparison among age groups, children demonstrated higher SEM% values (3.41–9.98%) than adolescents (2.81–9.08%) in both tests, indicating that adolescents presented slightly better reliability than children. The performance in the sit and reach and back scratch tests per trial (mean ± standard deviation), as well as the relative (ICC with 95% CI) and absolute (SEM, SEM%) reliability indices, for girl non-athletes are presented in Table 8.

### 3.2. Intersession Reliability

#### 3.2.1. Wrestling Athletes

##### Boys

Single trial. The test and retest sit and reach and back scratch values (mean and SD), as well as the relative and absolute reliability indices (ICC, SEM, SEM%, 95% LOA), for boy wrestling athletes (per age group) are presented in Table 9. The paired *t*-tests demonstrated non-significant differences between test and retest values in the sit and reach test and back scratch test in children (*p* = 0.16–0.45) and adolescents (*p* = 0.20–0.66). The systematic bias ranged from −0.07 to +0.52 cm in children and from −0.20 to +0.28 cm in adolescent boys. The relative and absolute reliability values between tests and retests were high for children in the sit and reach test (ICC = 0.997; SEM% = 2.33) and back scratch test (ICC = 0.998; SEM% = 4.34–5.61). Moreover, adolescents showed high relative reliability and absolute reliability in the sit and reach test (ICC = 0.998; SEM% = 1.64) and back scratch test (ICC = 0.998; SEM% = 2.67–4.27). Concerning the comparison among age groups, children demonstrated higher SEM% values (2.33–5.61%) than adolescents (1.64–4.27%) in both tests, indicating that adolescents presented better reliability than children.

Best or mean of three testing trials. The test and retest sit and reach and back scratch values (mean and SD), as well as the relative and absolute reliability indices (ICC, SEM, SEM%, 95% LOA), for boy wrestling athletes (per age group) are presented in Table 9. The paired *t*-tests demonstrated non-significant differences between the test and retest values in the sit and reach test and back scratch test in children (*p* = 0.18–0.55) and adolescents (*p* = 0.30–0.70). The systematic bias ranged from −0.05 to +0.50 cm in children and from −0.20 to +0.28 cm in adolescent boys. The relative and absolute reliability values between tests and retests were high for children in the sit and reach test (ICC = 0.997; SEM% = 2.32–2.37) and back scratch test (ICC = 0.998; SEM% = 2.33–5.60). Moreover, adolescents showed high relative reliability and absolute reliability in the sit and reach test (ICC = 0.998; SEM% = 1.63–1.66) and back scratch test (ICC = 0.998–0.999; SEM% = 2.60–3.97). Regarding the comparison among age groups, children demonstrated higher SEM% values (2.32–5.60%) than adolescents (1.63–3.97%) in both tests, indicating that adolescents presented better reliability than children.

##### Girls

Single trial. The test and retest sit and reach and back scratch values (mean and SD), as well as the relative and absolute reliability indices (ICC, SEM, SEM%, 95% LOA), for girl wrestling athletes (per age group) are presented in Table 10. The paired *t*-tests demonstrated non-significant differences between the test and retest values in the sit and reach test and back scratch test in children (*p* = 0.15–0.36) and adolescents (*p* = 0.21–0.40). The systematic bias ranged from −0.4 to +0.9 cm in children and from −0.19 to +0.4 cm in adolescent girls. The relative and absolute reliability values between tests and retests were high for children in the sit and reach test (ICC = 0.989; SEM% = 2.56) and back scratch test (ICC = 0.990–0.994; SEM% = 4.40–6.25). Moreover, adolescents showed high relative reliability and absolute reliability in the sit and reach test (ICC = 0.995; SEM% = 1.79) and back scratch test (ICC = 0.994–0.996; SEM% = 3.31–4.32). Regarding the comparison among age groups, children demonstrated higher SEM% values (2.56–6.25%) than adolescents (1.79–4.32%) in both tests, indicating that adolescents presented better reliability than children.

Best or mean of three testing trials. The test and retest sit and reach and back scratch values (mean and SD), as well as the relative and absolute reliability indices (ICC, SEM, SEM%, 95% LOA), for girl wrestling athletes (per age group) are presented in Table 10. The paired *t*-tests demonstrated non-significant differences between the test and retest values in the sit and reach test and back scratch test in children (*p* = 0.23–0.42) and adolescents (*p* = 0.35–0.75). The systematic bias ranged from −0.15 to +0.69 cm in children and from −0.30 to +0.36 cm in adolescent girls. The relative and absolute reliability values between tests and retests were high for children in the sit and reach test (ICC = 0.990; SEM% = 2.42–2.43) and back scratch test (ICC = 0.991–0.994; SEM% = 4.26–6.48). Moreover, adolescents showed high relative reliability and absolute reliability in the sit and reach test (ICC = 0.995–0.996; SEM% = 1.73–1.75) and back scratch test (ICC = 0.994–0.996; SEM% = 3.28–4.20). Regarding the comparison among age groups, children demonstrated higher SEM% values (2.42–6.48%) than adolescents (1.73–4.20%) in both tests, indicating that adolescents presented better reliability than children.

#### 3.2.2. Taekwondo

##### Boys

Single trial. The test and retest sit and reach and back scratch values (mean and SD), as well as the relative and absolute reliability indices (ICC, SEM, SEM%, 95% LOA), for boy taekwondo athletes (per age group) are presented in Table 11. The paired *t*-tests demonstrated non-significant differences between the test and retest values in the sit and reach test and back scratch test in children (*p* = 0.22–0.36) and adolescents (*p* = 0.36–0.55). The systematic bias ranged from −0.06 to +0.53 cm in children and from −0.45 to +0.24 cm in adolescent boys. The relative and absolute reliability values between tests and retests were high for children in the sit and reach test (ICC = 0.997; SEM% = 2.67) and back scratch test (ICC = 0.997–0.998; SEM% = 4.38–6.26). Moreover, adolescents showed high relative reliability and absolute reliability in the sit and reach test (ICC = 0.998; SEM% = 1.92) and back scratch test (ICC = 0.998; SEM% = 3.69–4.62). Regarding the comparison among age groups, children demonstrated higher SEM% values (2.67–6.26%) than adolescents (1.92–4.62%) in both tests, indicating that adolescents presented better reliability than children.

Best or mean of three testing trials. The test and retest sit and reach and back scratch values (mean and SD), as well as the relative and absolute reliability indices (ICC, SEM, SEM%, 95% LOA), for boy taekwondo athletes (per age group) are presented in Table 11. The paired *t*-tests demonstrated non-significant differences between the test and retest values in the sit and reach test and back scratch test in children (*p* = 0.32–0.57) and adolescents (*p* = 0.51–0.73). The systematic bias ranged from −0.06 to +0.44 cm in children and from −0.10 to +0.11 cm in adolescent boys. The relative and absolute reliability values between tests and retests were high for children in the sit and reach test (ICC = 0.997; SEM% = 2.67–2.82) and back scratch test (ICC = 0.996–0.998; SEM% = 4.41–6.55). Moreover, adolescents showed high relative reliability and absolute reliability in the sit and reach test (ICC = 0.998; SEM% = 1.87–1.97) and back scratch test (ICC = 0.998; SEM% = 3.54–4.98). Regarding the comparison among age groups, children demonstrated higher SEM% values (2.67–6.55%) than adolescents (1.87–4.98%) in both tests, indicating that adolescents presented better reliability than children.

##### Girls

Single trial. The test and retest sit and reach and back scratch values (mean and SD), as well as the relative and absolute reliability indices (ICC, SEM, SEM%, 95% LOA), for girl taekwondo athletes (per age group) are presented in Table 12. The paired *t*-tests demonstrated non-significant differences between the test and retest values in the sit and reach test and back scratch test in children (*p* = 0.23–0.43) and adolescents (*p* = 0.39–0.58). The systematic bias ranged from −0.24 to +0.15 cm in children and from −0.05 to +0.37 cm in adolescent girls. The relative and absolute reliability values between tests and retests were high for children in the sit and reach test (ICC = 0.996; SEM% = 1.83) and back scratch test (ICC = 0.997; SEM% = 4.42–6.72). Moreover, adolescents showed high relative reliability and absolute reliability in the sit and reach test (ICC = 0.998; SEM% = 1.16) and back scratch test (ICC = 0.996–0.998; SEM% = 3.50–4.46). Regarding the comparison among age groups, children demonstrated higher SEM% values (1.83–6.72%) than adolescents (1.16–4.46%) in both tests, indicating that adolescents presented better reliability than children.

Best or mean of three testing trials. The test and retest sit and reach and back scratch values (mean and SD), as well as the relative and absolute reliability indices (ICC, SEM, SEM%, 95% LOA), for girl taekwondo athletes (per age group) are presented in Table 12. The paired *t*-tests demonstrated non-significant differences between the test and retest values in the sit and reach test and back scratch test in children (*p* = 0.44–0.66) and adolescents (*p* = 0.53–0.74). The systematic bias ranged from −0.22 to +0.13 cm in children and from −0.12 to +0.17 cm in adolescent girls. The relative and absolute reliability values between tests and retests were high for children in the sit and reach test (ICC = 0.995–0.996; SEM% = 2.03–2.09) and back scratch test (ICC = 0.996–0.998; SEM% = 4.98–6.76). Moreover, adolescents showed high relative reliability and absolute reliability in the sit and reach test (ICC = 0.997; SEM% = 1.39–1.40) and back scratch test (ICC = 0.996–0.998; SEM% = 2.71–4.97). Regarding the comparison among age groups, children demonstrated higher SEM% values (2.03–6.76%) than adolescents (1.39–4.97%) in both tests, indicating that adolescents presented better reliability than children.

#### 3.2.3. Non-Athletes

##### Boys

Single trial. The test and retest sit and reach and back scratch values (mean and SD), as well as the relative and absolute reliability indices (ICC, SEM, SEM%, 95% LOA), for boy non-athletes (per age group) are presented in Table 13. The paired *t*-tests demonstrated non-significant differences between the test and retest values in the sit and reach test and back scratch test in children (*p* = 0.23–0.43) and adolescents (*p* = 0.36–0.57). The systematic bias ranged from −0.09 to −0.3 cm in children and from −0.06 to +0.19 cm in adolescent boys. The relative and absolute reliability values between tests and retests were good to high for children in the sit and reach test (ICC = 0.994; SEM% = 4.93) and back scratch test (ICC = 0.993–0.997; SEM% = 7.70–9.39). Moreover, adolescents showed high relative reliability and absolute reliability in the sit and reach test (ICC = 0.997; SEM% = 2.99) and back scratch test (ICC = 0.996–0.998; SEM% = 5.01–8.22). Regarding the comparison among age groups, children demonstrated higher SEM% values (4.93–9.39%) than adolescents (2.99–8.22%) in both tests, indicating that adolescents presented better reliability than children.

Best or mean of three testing trials. The test and retest sit and reach and back scratch values (mean and SD), as well as the relative and absolute reliability indices (ICC, SEM, SEM%, 95% LOA), for boy non-athletes (per age group) are presented in Table 13. The paired *t*-tests demonstrated non-significant differences between the test and retest values in the sit and reach test and back scratch test in children (*p* = 0.37–0.59) and adolescents (*p* = 0.55–0.74). The systematic bias ranged from −0.10 to −0.03 cm in children and from −0.06 to +0.07 cm in adolescent boys. The relative and absolute reliability values between tests and retests were good to high for children in the sit and reach test (ICC = 0.994–0.995; SEM% = 4.23–4.56) and back scratch test (ICC = 0.995–0.997; SEM% = 7.05–9.66). Additionally, adolescents showed high relative reliability and absolute reliability in the sit and reach test (ICC = 0.997; SEM% = 2.96–2.98) and back scratch test (ICC = 0.996–0.998; SEM% = 5.00–8.67). Concerning the comparison among age groups, children demonstrated higher SEM% values (2.96–8.67%) than adolescents (2.99–8.22%) in both tests, indicating that adolescents presented better reliability than children.

##### Girls

Single trial. The test and retest sit and reach and back scratch values (mean and SD), as well as the relative and absolute reliability indices (ICC, SEM, SEM%, 95% LOA), for girl non-athletes (per age group) are presented in Table 14. The paired *t*-tests demonstrated non-significant differences between the test and retest values in the sit and reach test and back scratch test in children (*p* = 0.21–0.49) and adolescents (*p* = 0.33–0.59). The systematic bias ranged from +0.06 to +0.3 cm in children and from −0.09 to +0.5 cm in adolescent girls. The relative and absolute reliability values between tests and retests were good to high for children in the sit and reach test (ICC = 0.995; SEM% = 3.79) and back scratch test (ICC = 0.998; SEM% = 9.90–9.98). Moreover, adolescents showed good to high relative reliability and absolute reliability in the sit and reach test (ICC = 0.996; SEM% = 3.27) and back scratch test (ICC = 0.997–0.998; SEM% = 7.03–8.40). Regarding the comparison among age groups, children demonstrated higher SEM% values (3.79–9.98%) than adolescents (3.27–8.40%) in both tests, indicating that adolescents presented better reliability than children.

Best or mean of three testing trials. The test and retest sit and reach and back scratch values (mean and SD), as well as the relative and absolute reliability indices (ICC, SEM, SEM%, 95% LOA), for girl non-athletes (per age group) are presented in Table 14. The paired *t*-tests demonstrated non-significant differences between the test and retest values in the sit and reach test and back scratch test in children (*p* = 0.40–0.69) and adolescents (*p* = 0.60–0.76). The systematic bias ranged from −0.05 to +0.53 cm in children and from −0.08 to +0.35 cm in adolescent girls. The relative and absolute reliability values between tests and retests were good to high for children in the sit and reach test (ICC = 0.996; SEM% = 3.40–3.41) and back scratch test (ICC = 0.998; SEM% = 9.52–9.95). Likewise, adolescents showed good to high relative reliability and absolute reliability in the sit and reach test (ICC = 0.996–0.998; SEM% = 3.19–3.24) and back scratch test (ICC = 0.997–0.998; SEM% = 7.13–8.60). Concerning the comparison among age groups, children demonstrated higher SEM% values (3.40–9.95%) than adolescents (3.19–8.60%) in both tests, indicating that adolescents presented better reliability than children.

## 4. Discussion

The novel aspect of this study is that it examined the effects of sport activities (wrestling, taekwondo, no participation in organized physical activity), age (children, adolescents), and sex (boys, girls) on the intrasession and intersession reliability (using various absolute and relative reliability indices) of flexibility measurements (sit and reach test, back scratch test). This study also investigated whether the number of testing trials and the method used for the determination of performance in flexibility tests (single trial, best of three trials, or average of three trials) affected their reliability. The results showed that upper- and lower-body flexibility, using the sit and reach and the back scratch tests, could be reliably assessed in boys and girls during the developmental years. It should be, however, mentioned that children displayed slightly lower intrasession and intersession reliability (higher SEM% values) compared to adolescents, irrespective of the sport activity and sex. Additionally, non-athletes exhibited slightly lower intrasession and intersession reliability (higher SEM% values) compared to taekwondo and wrestling athletes, irrespective of age and sex. Furthermore, it seems that a single trial and the best and average of three testing trials are equally reliable for flexibility measurement, irrespective of the sport, age, and sex.

Generally, the findings of the present study are in line with previous studies that have examined the reliability of the sit and reach test in untrained children and adolescents or young athletes [25,27,28], demonstrating high reliability (ICC = 0.93–0.99). Additionally, the results of the present study are consistent with previous studies that have shown good to high reliability (ICC = 0.88–0.93) for the shoulder stretch test in untrained children and adolescents [26] and high reliability (ICC = 0.99–1) in young tennis players [28]. However, it should be mentioned that different factors, such as the subjects’ characteristics and the testing protocol, may affect the reliability of measurements.

An important factor that could affect the reliability of measurements is age. In the present study, we observed higher SEM% values in children athletes and non-athletes compared to adolescent athletes and non-athletes for both flexibility tests (sit and reach test, back scratch test). In the scientific literature, there is limited information regarding the age effect on the reliability of flexibility measurements during the developmental years. Our findings are in line with a previous study [41] in young soccer players (11–19 years old) that showed different reliability values among age groups. In more detail, Díaz-Escobar et al. [41] showed that older age groups from sub-13 to sub-18 presented higher Cronbach’s alpha values of 0.80–0.94 than the younger age group, i.e., sub-11 (Cronbach’s alpha = 0.72), during the modified Thomas flexibility test. Furthermore, our findings are consistent with previous studies [38,39,52] that examined other physical fitness parameters (e.g., handgrip strength, cervical strength) and found significant age effects on reliability during the developmental years, indicating higher reliability with increasing age. For example, Svenson et al. [39] reported that handgrip strength measurements (using the best of three trials) were more reliable in a 14-year-old group (ICC = 0.96, SEM%: 5.2) than in a 10-year-old group (ICC = 0.78, SEM%: 12.5). Additionally, Gerodimos and Karatrantou [53] demonstrated that prepubertal wrestlers showed greater SEM% values than pubertal wrestlers regarding handgrip strength, and Batatolis et al. [52] showed greater SEM% values in prepubertal boys (SEM% = 5.82–8.62) compared to pubertal boys (SEM% = 3.8–5.5) regarding cervical strength measurements, indicating that pubertal boys showed slightly higher reliability than prepubertal boys. Previous studies that have observed significant age effects on the reliability of measurements mention that these differences among age groups may be attributed to different factors, such as mood, motivation, attention between testing occasions, learning effects, the maturity of the nervous system, and biomechanical factors [38,39]. It may be argued that neural maturation during the developmental years [42] (from childhood to adolescence) could impact the overall level of performance, as well as its reliability, leading to changes in the ability of individuals to focus on the task (increased cognitive control and attention in adolescents vs. children). Furthermore, participation in physical activities and sports may positively affect neural maturation. Specifically, compared to non-athletes, due to neuroplastic changes in the brain as a result of specialized training, athletes have better motor control, cognitive processing, and sensory perception [54], influencing their performance levels and, as a result, the reliability of measurements.

Additional factors that could affect the reliability of measurements are engagement in sports activities and physical fitness levels. Our study demonstrated better absolute reliability (lower SEM% values) in wrestling and taekwondo athletes (children: ICC = 0.988–0.998, SEM% = 2.31–7.44; adolescents: ICC = 0.993–0.999, SEM% = 1.13–5.19) compared to non-athletes (children: ICC = 0.992–0.997, SEM% = 3.40–9.98; adolescents: ICC = 0.996–0.998, SEM% = 2.81–8.94) for both flexibility tests (sit and reach test, back scratch test). To the best of our knowledge, in the context of the developmental years, no previous study has examined the differences in the reliability of flexibility measurements between athletes and non-athletes. However, regarding other physical fitness parameters, such as strength and power, there is adequate information with which to compare our results, using cross-parameter comparisons. Strength and power, as well as flexibility, are interconnected neuromuscular performance parameters and could potentially behave similarly in the context of test–retest reliability. Indeed, previous studies on other physical fitness parameters (i.e., strength and power) showed better reliability in trained vs. untrained participants [55], as well as between athletes and non-athletes [37,40], reinforcing our findings in the flexibility test. Previous studies that found significant differences in reliability between athletes and non-athletes mentioned that, as the process of neural adaptation occurs secondary to the training experience, the ability to engage motor fibers and the reliability of measurements are enhanced. The reliability of measurements may also be influenced by differences in performance levels. Three previous studies [25,43,56] have demonstrated that the performance level in terms of flexibility affects the reliability of measurements. Indeed, Ramirez et al. [25], in Colombian boys and girls (9–17.9 years old), indicated that the worse the performance in the sit and reach test, the worse the degree of agreement. Moreover, Ortega et al. [43] observed that adolescents who scored highly in the back-saver sit and reach test had better intertrial agreement compared with adolescents who obtained lower scores, indicating that the flexibility level can affect its reliability.

Regarding the sex effect on the reliability of measurements, in the present study, we observed no differences in reliability between boys and girls (irrespective of sport activity and age) in both the sit and reach and back scratch tests. In the same context, Luna-Villouta et al. [28] showed similar ICC values in young tennis players (14–16 years old) among boys and girls in both the sit and reach test (ICC = 1.00 in boys and ICC = 0.99 in girls) and the shoulder stretch test (ICC = 0.99 in boys and ICC = 1.00 in girls). Meanwhile, Ortega et al. [43], during the back-saver sit and reach test, found a borderline significant sex difference. In the above study of Ortega et al. [43], adolescent girls showed a greater intertrial difference (1.4 cm) than adolescent boys (−0.3 cm) in the back-saver sit and reach test.

Finally, the number of testing trials performed (i.e., one vs. three), as well as the different methods used to determine performance (average vs. best value), may potentially affect the reliability of measurements. In our study, we found that a single trial and the best or mean of three testing trials were equally reliable during flexibility measurements (sit and reach test, back scratch tests) in boys and girls, as well as in athletes and non-athletes, during the developmental years (children and adolescents). Thus, a testing protocol with one trial may be used as an equally reliable, less fatiguing, and less time-consuming protocol for the evaluation of flexibility in young healthy individuals, compared to a testing protocol with three testing trials (best or average value). Although, regarding flexibility tests, there is no information regarding the reliability of measurements when using different numbers of trials, for other physical fitness parameters (e.g., handgrip strength), there is adequate information, albeit with conflicting results. On the one hand, Mathiowetz et al. [44], Svensson et al. [39], and Karatrantou et al. [45] reported that the mean of three testing trials had higher test–retest reliability than either a single trial or the best of three trials in healthy individuals [39,44] and in individuals with intellectual disabilities [45]. On the other hand, other studies have observed that a single trial and the mean or the best of two or three trials are equally reliable in determining the maximal handgrip strength in healthy individuals, as well as in individuals who have undergone carpal tunnel decompression or flexor tendon repair [46,47,48]. Future studies are needed to draw more valid conclusions regarding the effects of testing trials on reliability, especially in flexibility measurements, where the literature is inadequate.

The present study has some limitations that could affect the generalization of its findings. First of all, the results of the present study are limited to children and adolescent athletes (wrestling or taekwondo) and non-athletes, and they cannot be generalized to other target population groups. Future studies could examine and create indicative–normative values for flexibility performance in individuals of other age groups, sports, or physical activity levels. Furthermore, the results of the present study are limited to the two flexibility tests (sit and reach and back scratch) that were used. However, future studies could examine and compare the reliability of other flexibility tests (field or laboratory) that are used in the scientific literature, as well as evaluating flexibility performance in other muscle groups of the human body. Other limitations of the present study are the measurement time and the warm-up protocol used, which could affect flexibility performance. Future studies could examine and compare flexibility performance and therefore its reliability using different measurement times and warm-up protocols.

## 5. Conclusions

In conclusion, the results of the present study suggest that the sit and reach and back scratch tests are reliable tests to assess flexibility in boy and girl athletes (wrestling and taekwondo) and non-athletes during the developmental years (children and adolescents). It seems, however, that engagement in sports and age could affect the reliability of these measurements. Specifically, it should be mentioned that athletes (wrestling and taekwondo athletes) present slightly better reliability compared to non-athletes. Additionally, adolescents present slightly better reliability compared to children. We also observed that sex did not affect the reliability of the sit and reach and back scratch tests. Finally, we observed similar test–retest reliability when using either a single trial or the best/mean of three testing trials. The results of the present study have important practical implications for coaches and physical fitness trainers, who could reliably use these tests (sit and reach and back scratch) for flexibility monitoring and training planning, as well as for injury prevention and rehabilitation. However, better familiarization, before the main testing, is important in children compared to adolescents and in non-athletes compared to athletes for more reliable results. Finally, it seems that, during measurement, a testing protocol with one trial could be used as an equally reliable, less fatiguing, and less time-consuming approach for the evaluation of flexibility, compared to a testing protocol with three trials.

## Figures and Tables

**Table 1:** Age and anthropometric and training characteristics of the participants per sport activity, age, and sex (mean ± standard deviation).

Variable	Age Group	Wrestling Athletes (n = 80)	Taekwondo Athletes (n = 80)	Non-Athletes (n = 80)
Age (years old)	Children	Boys: 8.77 ± 0.73 Girls: 8.84 ± 0.77	Boys: 8.58 ± 0.74 Girls: 8.70 ± 0.74	Boys: 8.90 ± 0.75 Girls: 8.69 ± 0.73
	Adolescents	Boys: 13.88 ± 0.80 * Girls: 13.52 ± 0.83 *	Boys: 13.75 ± 0.84 * Girls: 13.67 ± 0.81 *	Boys: 13.62 ± 0.75 * Girls: 13.44 ± 0.66 *
Body height (cm)	Children	Boys: 140.02 ± 10.04 Girls: 136.53 ± 7.37	Boys: 135.61 ± 7.49 Girls: 138.87 ± 9.49	Boys: 142.49 ± 8.08 Girls: 142.07 ± 9.62
	Adolescents	Boys: 162.62 ± 9.18 * Girls: 156.64 ± 9.06 *	Boys: 166.50 ± 8.72 * Girls: 158.59 ± 10.04 *	Boys: 163.76 ± 10.73 * Girls: 162.35 ± 8.63 *
Body mass (kg)	Children	Boys: 38.19 ± 8.07 Girls: 34.63 ± 7.64	Boys: 36.78 ± 10.88 Girls: 36.87 ± 13.42	Boys: 37.69 ± 6.75 Girls: 39.23 ± 10.14
	Adolescents	Boys: 57.70 ± 8.12 * Girls: 52.62 ± 11.36 *	Boys: 55.59 ± 10.35 * Girls: 48.00 ± 8.06 *	Boys: 52.86 ± 11.05 * Girls: 52.65 ± 7.27 *
Training age (years old)	Children	Boys: 2.58 ± 1.62 Girls: 2.43 ± 1.46	Boys: 2.55 ± 1.31 Girls: 2.40 ± 1.30	Boys: - Girls: -
	Adolescents	Boys: 5.00 ± 2.63 * Girls: 4.63 ± 1.96 *	Boys: 4.80 ± 2.11 * Girls: 4.68 ± 2.05 *	Boys: - Girls: -
Training frequency (times/week)	Children	Boys: 3.45 ± 0.40 Girls: 3.35 ± 0.10	Boys: 3.40 ± 0.30 Girls: 3.30 ± 0.20	Boys: - Girls: -
	Adolescents	Boys: 4.10 ± 0.40 * Girls: 3.90 ± 0.55 *	Boys: 4.05 ± 0.45 * Girls: 3.85 ± 0.50 *	Boys: - Girls: -

* *p* = 0.02–0.01, indicating a statistically significant difference between children and adolescent wrestling athletes, between children and adolescent taekwondo athletes, and between children and adolescent non-athletes.

**Table 2:** Flexibility tests.

Sit and reach test	
Measured Index: Lumbar spine and hamstring flexibility.	
Equipment: Flex-Tester box (Novel Products Inc., Rockton, IL, USA).	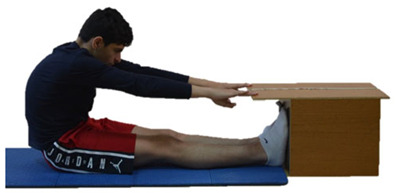
Description: From a sitting position, with the knees extended and the soles (without shoes) touching the box, the participant leaned forward slowly as far as possible, while exhaling, without bending his/her knees. The participant remained in the final position for at least 2 s [20].	
Testing protocol: Three maximal trials were performed, with an interval of 15 s/trial.	
Evaluation: The performance of each trial in centimeters (cm) was scored. In the event that the participant’s fingers did not reach the box, the measurement was negative; otherwise, it was positive.	
Back scratch test (also called zipper test or shoulder stretch test)	
Measured Index: Range of motion of the shoulder joint.	
Equipment: Measuring tape.	
Description: The participant, from a standing position (trunk straight, legs together and extended), with the arms placed behind the back, tried to bring his/her fingers of the hands closer. The test was performed at a slow pace, and the participant remained in the final position for 2 s. During the test, the fingers of the hands had to be outstretched and the trunk upright [2]. The test was performed in both hands (preferred and non-preferred hand). Hand preference was determined by asking the participant which hand was used to hold a pencil.	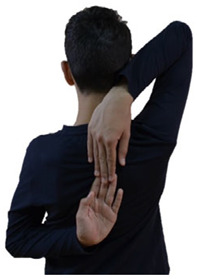
Testing protocol: Three maximal trials were performed for each hand, with an interval of 30 s/trial.	
Evaluation: The distance between the tips of the middle fingers of the two hands was recorded in centimeters (cm): (a) if the fingertips did not touch each other, the score was negative; (b) if they touched each other, the score was zero; and (c) if they overlapped, the score was positive.	

**Table 3:** Performance in flexibility tests per trial (mean ± standard deviation), as well as relative and absolute reliability indices, in boy wrestling athletes per age group.

Children							
	Trial 1	Trial 2	Trial 3	ICC	95% CI	SEM	SEM%
Sit and reach test	16.98 ± 7.46 cm	16.82 ± 7.42 cm	16.40 ± 7.30 cm	0.997	0.987–0.999	0.40 cm	2.42
Back scratch test—PH	2.09 ± 2.21 cm	2.04 ± 2.10 cm	1.98 ± 2.00 cm	0.998	0.994–0.999	0.09 cm	4.62
Back scratch test—NPH	1.12 ± 1.32 cm	0.97 ± 1.30 cm	1.00 ± 1.29 cm	0.998	0.994–0.999	0.06 cm	5.66
Adolescents							
	Trial 1	Trial 2	Trial 3	ICC	95% CI	SEM	SEM%
Sit and reach test	19.72 ± 7.27 cm	19.8 ± 7.4 cm	19.5 ± 7.44 cm	0.998	0.996–0.999	0.33 cm	1.67
Back scratch test—PH	2.20 ± 1.5 cm	2.28 ± 1.45 cm	2.23 ± 1.5 cm	0.998	0.995–0.999	0.06 cm	2.57
Back scratch test—NPH	1.47 ± 1.39 cm	1.52 ± 1.42 cm	1.62 ± 1.42 cm	0.998	0.994–0.999	0.06 cm	4.10

ICC: intraclass correlation coefficient, 95% CI: 95% confidence interval, SEM: standard error of measurement, PH: preferred hand, NPH: non-preferred hand.

**Table 4:** Performance in flexibility tests per trial (mean ± standard deviation), as well as relative and absolute reliability indices, in girl wrestling athletes per age group.

Children							
	Trial 1	Trial 2	Trial 3	ICC	95% CI	SEM	SEM%
Sit and reach test	19.60 ± 4.57 cm	19.59 ± 4.83 cm	19.51 ± 5.11 cm	0.988	0.974–0.995	0.53 cm	2.71
Back scratch test—PH	5.60 ± 2.65 cm	5.68 ± 2.60 cm	5.79 ± 2.70 cm	0.990	0.980–0.998	0.27 cm	4.66
Back scratch test—NPH	4.25 ± 5.48 cm	4.50 ± 5.58 cm	4.36 ± 5.49 cm	0.993	0.986–0.999	0.29 cm	6.73
Adolescents							
	Trial 1	Trial 2	Trial 3	ICC	95% CI	SEM	SEM%
Sit and reach test	24.20 ± 6.33 cm	24.36 ± 6.60 cm	24.91 ± 6.80 cm	0.995	0.989–0.998	0.47 cm	1.90
Back scratch test—PH	6.69 ± 3.91 cm	6.72 ± 3.00 cm	7.00 ± 3.36 cm	0.995	0.986–0.999	0.24 cm	3.56
Back scratch test—NPH	4.79 ± 2.88 cm	4.80 ± 2.87 cm	4.60 ± 2.06 cm	0.993	0.985–0.998	0.22 cm	4.60

ICC: intraclass correlation coefficient, 95% CI: 95% confidence interval, SEM: standard error of measurement, PH: preferred hand, NPH: non-preferred hand.

**Table 5:** Performance in flexibility tests per trial (mean ± standard deviation), as well as relative and absolute reliability indices, in boy taekwondo athletes per age group.

Children							
	Trial 1	Trial 2	Trial 3	ICC	95% CI	SEM	SEM%
Sit and reach test	14.47 ± 7.36 cm	14.80 ± 7.67 cm	14.13 ± 7.42 cm	0.998	0.994–0.999	0.33 cm	2.31
Back scratch test—PH	2.84 ± 2.39 cm	2.69 ± 2.35 cm	2.70 ± 2.95 cm	0.996	0.990–0.998	0.16 cm	5.90
Back scratch test—NPH	0.95 ± 1.50 cm	0.93 ± 1.55 cm	1.00 ± 1.40 cm	0.998	0.997–0.999	0.07 cm	6.91
Adolescents							
	Trial 1	Trial 2	Trial 3	ICC	95% CI	SEM	SEM%
Sit and reach test	14.86 ± 6.58 cm	15.23 ± 6.72 cm	14.92 ± 6.73 cm	0.998	0.995–0.999	0.30 cm	1.99
Back scratch test—PH	6.65 ± 5.60 cm	6.69 ± 5.49 cm	6.31 ± 5.49 cm	0.998	0.994–0.999	0.25 cm	3.77
Back scratch test—NPH	2.68 ± 3.06 cm	2.73 ± 3.01 cm	2.72 ± 3.00 cm	0.998	0.996–0.999	0.14 cm	4.98

ICC: intraclass correlation coefficient, 95% CI: 95% confidence interval, SEM: standard error of measurement, PH: preferred hand, NPH: non-preferred hand.

**Table 6:** Performance in flexibility tests per trial (mean ± standard deviation), as well as relative and absolute reliability indices, in girl taekwondo athletes per age group.

Children							
	Trial 1	Trial 2	Trial 3	ICC	95% CI	SEM	SEM%
Sit and reach test	19.94 ± 5.60 cm	20.07 ± 5.63 cm	20.05 ± 5.61 cm	0.996	0.992–0.998	0.36 cm	1.77
Back scratch test—PH	3.26 ± 2.63 cm	3.12 ± 3.62 cm	3.32 ± 2.58 cm	0.997	0.994–0.999	0.16 cm	4.99
Back scratch test—NPH	1.80 ± 2.60 cm	1.71 ± 2.07 cm	1.84 ± 2.60 cm	0.997	0.996–0.999	0.13 cm	7.44
Adolescents							
	Trial 1	Trial 2	Trial 3	ICC	95% CI	SEM	SEM%
Sit and reach test	24.05 ± 6.04 cm	24.21 ± 6.06 cm	23.94 ± 6.07 cm	0.998	0.995–0.999	0.27 cm	1.13
Back scratch test—PH	6.53 ± 4.03 cm	6.77 ± 4.02 cm	6.73 ± 4.06 cm	0.996	0.993–0.998	0.26 cm	3.82
Back scratch test—NPH	3.51 ± 4.04 cm	3.59 ± 4.00 cm	3.29 ± 4.02 cm	0.998	0.996–0.999	0.18 cm	5.19

ICC: intraclass correlation coefficient, 95% CI: 95% confidence interval, SEM: standard error of measurement, PH: preferred hand, NPH: non-preferred hand.

**Table 7:** Performance in flexibility tests per trial (mean ± standard deviation), as well as relative and absolute reliability indices, in boy non-athletes per age group.

Children							
	Trial 1	Trial 2	Trial 3	ICC	95% CI	SEM	SEM%
Sit and reach test	9.63 ± 5.68 cm	9.73 ± 5.53 cm	9.28 ± 5.62 cm	0.994	0.984–0.997	0.43 cm	4.55
Back scratch test—PH	2.04 ± 1.87 cm	1.85 ± 1.88 cm	1.74 ± 1.88 cm	0.992	0.983–0.997	0.17 cm	8.94
Back scratch test—NPH	−0.20 ± 0.70 cm	−0.33 ± 0.70 cm	−0.62 ± 0.65 cm	0.997	0.990–0.998	0.04 cm	9.76
Adolescents							
	Trial 1	Trial 2	Trial 3	ICC	95% CI	SEM	SEM%
Sit and reach test	9.71 ± 5.22 cm	9.98 ± 5.19 cm	9.62 ± 5.19 cm	0.997	0.989–0.999	0.28 cm	2.92
Back scratch test—PH	2.05 ± 1.43 cm	1.90 ± 1.44 cm	1.80 ± 1.43 cm	0.996	0.988–0.998	0.09 cm	4.73
Back scratch test—NPH	−0.17 ± 0.38 cm	−0.18 ± 0.4 cm	−0.19 ± 0.3 cm	0.998	0.992–0.999	0.02 cm	8.94

ICC: intraclass correlation coefficient, 95% CI: 95% confidence interval, SEM: standard error of measurement, PH: preferred hand, NPH: non-preferred hand.

**Table 8:** Performance in flexibility tests per trial (mean ± standard deviation), as well as relative and absolute reliability indices, in girl non-athletes per age group.

Children							
	Trial 1	Trial 2	Trial 3	ICC	95% CI	SEM	SEM%
Sit and reach test	18.30 ± 9.86 cm	18.08 ± 9.68 cm	18.06 ± 9.85 cm	0.996	0.994–0.999	0.62 cm	3.41
Back scratch test—PH	1.77 ± 4.01 cm	1.97 ± 4.04 cm	1.80 ± 4.09 cm	0.998	0.995–0.999	0.18 cm	9.79
Back scratch test—NPH	−1.61 ± 3.60 cm	−1.68 ± 3.70 cm	−1.55 ± 3.50 cm	0.998	0.996–0.999	0.16 cm	9.98
Adolescents							
	Trial 1	Trial 2	Trial 3	ICC	95% CI	SEM	SEM%
Sit and reach test	18.50 ± 9.66 cm	18.56 ± 9.44 cm	18.51 ± 9.42 cm	0.997	0.995–0.999	0.52 cm	2.81
Back scratch test—PH	5.34 ± 7.10 cm	5.58 ± 7.17 cm	5.33 ± 7.02 cm	0.997	0.994–0.999	0.39 cm	7.18
Back scratch test—NPH	1.48 ± 3.01 cm	1.52 ± 3.03 cm	1.46 ± 3.02 cm	0.998	0.996–0.999	0.14 cm	9.08

ICC: intraclass correlation coefficient, 95% CI: 95% confidence interval, SEM: standard error of measurement, PH: preferred hand, NPH: non-preferred hand.

**Table 9:** Test–retest values (mean ± SD), as well as absolute and relative reliability indices, in boy wrestling athletes (per age group).

Children							
	Test	Retest	ICC	SEM	SEM%	95% LOA Lower	95% LOA Upper
Single trial							
Sit and reach test	16.98 ± 7.46 cm	17.50 ± 7.20 cm	0.997	0.40 cm	2.33	−1.83 cm	2.87 cm
Back scratch test—PH	2.09 ± 2.21 cm	2.25 ± 2.00 cm	0.998	0.09 cm	4.34	−2.29 cm	2.61 cm
Back scratch test—NPH	1.12 ± 1.32 cm	1.05 ± 1.40 cm	0.998	0.06 cm	5.61	−2.62 cm	2.48 cm
Best of three trials							
Sit and reach test	17.20 ± 7.50 cm	17.70 ± 7.30 cm	0.997	0.41 cm	2.32	−1.75 cm	2.75 cm
Back scratch test—PH	2.12 ± 2.18 cm	2.20 ± 2.00 cm	0.998	0.09 cm	4.33	−2.27 cm	2.43 cm
Back scratch test—NPH	1.15 ± 1.30 cm	1.10 ± 1.30 cm	0.998	0.06 cm	5.17	−2.70 cm	2.60 cm
Mean of three trials							
Sit and reach test	16.74 ± 7.39 cm	16.99 ± 7.2 cm	0.997	0.40 cm	2.37	−2.38 cm	2.88 cm
Back scratch test—PH	2.04 ± 2.10 cm	2.00 ± 2.02 cm	0.998	0.09 cm	4.57	−2.88 cm	2.81 cm
Back scratch test—NPH	1.03 ± 1.30 cm	1.09 ± 1.35 cm	0.998	0.06 cm	5.60	−2.99 cm	3.12 cm
Adolescents							
	Test	Retest	ICC	SEM	SEM%	95% LOA Lower	95% LOA Upper
Single trial							
Sit and reach test	19.72 ± 7.27 cm	20.00 ± 7.30 cm	0.998	0.33 cm	1.64	−2.27 cm	2.83 cm
Back scratch test—PH	2.20 ± 1.50 cm	2.00 ± 1.40 cm	0.998	0.06 cm	2.67	−2.94 cm	2.54 cm
Back scratch test—NPH	1.47 ± 1.39 cm	1.40 ± 1.35 cm	0.998	0.06 cm	4.27	−3.01 cm	2.87 cm
Best of three trials							
Sit and reach test	19.92 ± 7.30 cm	20.20 ± 7.30 cm	0.998	0.33 cm	1.63	−2.37 cm	2.93 cm
Back scratch test—PH	2.40 ± 1.70 cm	2.20 ± 1.5 cm	0.999	0.06 cm	2.69	−2.75 cm	2.35 cm
Back scratch test—NPH	1.57 ± 1.40 cm	1.50 ± 1.30 cm	0.998	0.06 cm	3.93	−2.91 cm	2.77 cm
Mean of three trials							
Sit and reach test	19.68 ± 7.37 cm	19.90 ± 7.30 cm	0.998	0.33 cm	1.66	−2.23 cm	2.67 cm
Back scratch test—PH	2.24 ± 1.48 cm	2.2 ± 1.5 cm	0.998	0.06 cm	2.60	−2.68 cm	2.61 cm
Back scratch test—NPH	1.54 ± 1.41 cm	1.48 ± 1.27 cm	0.998	0.06 cm	3.97	−2.90 cm	2.79 cm

ICC: intraclass correlation coefficient, 95% CI: 95% confidence interval, SEM: standard error of measurement, PH: preferred hand, NPH: non-preferred hand.

**Table 10:** Test–retest values (mean ± SD), as well as absolute and relative reliability indices, in girl wrestling athletes (per age group).

Children							
	Test	Retest	ICC	SEM	SEM%	95% LOA Lower	95% LOA Upper
Single trial							
Sit and reach test	19.6 ± 4.57 cm	20.05 ± 5.2 cm	0.989	0.51 cm	2.56	−1.84 cm	3.64 cm
Back scratch test—PH	5.6 ± 2.65 cm	5.2 ± 2.1 cm	0.990	0.24 cm	4.40	−3.54 cm	2.74 cm
Back scratch test—NPH	4.25 ± 3.48 cm	4.15 ± 3.3 cm	0.994	0.26 cm	6.25	−2.45 cm	2.25 cm
Best of three trials							
Sit and reach test	20.01 ± 4.6 cm	20.7 ± 5.3 cm	0.990	0.50 cm	2.43	−1.86 cm	3.24 cm
Back scratch test—PH	5.8 ± 2.55 cm	5.65 ± 2.6 cm	0.991	0.24 cm	4.27	−2.80 cm	2.50 cm
Back scratch test—NPH	4.45 ± 3.3 cm	4.35 ± 3.0 cm	0.994	0.24 cm	5.55	−2.84 cm	2.64 cm
Mean of three trials							
Sit and reach test	19.57 ± 4.84 cm	19.8 ± 4.7 cm	0.990	0.48 cm	2.42	−2.20 cm	2.66 cm
Back scratch test—PH	5.69 ± 2.65 cm	5.54 ± 2.7 cm	0.992	0.24 cm	4.26	−2.85 cm	2.55 cm
Back scratch test—NPH	4.37 ± 3.52 cm	4.3 ± 3.2 cm	0.993	0.28 cm	6.48	−2.91 cm	2.77 cm
Adolescents							
	Test	Retest	ICC	SEM	SEM%	95% LOA Lower	95% LOA Upper
Single trial							
Sit and reach test	24.2 ± 6.33 cm	24.6 ± 6 cm	0.995	0.44 cm	1.79	−2.15 cm	2.95 cm
Back scratch test—PH	6.69 ± 3.91 cm	6.5 ± 3 cm	0.996	0.22 cm	3.31	−3.33 cm	2.95 cm
Back scratch test—NPH	4.79 ± 2.88 cm	4.85 ± 2.5 cm	0.994	0.21 cm	4.32	−3.47 cm	3.59 cm
Best of three trials							
Sit and reach test	24.55 ± 6.25 cm	24.9 ± 6.01 cm	0.995	0.43 cm	1.75	−2.30 cm	3.00 cm
Back scratch test—PH	6.9 ± 3.9 cm	6.8 ± 3.2 cm	0.996	0.22 cm	3.28	−3.04 cm	2.84 cm
Back scratch test—NPH	4.84 ± 2.9 cm	4.9 ± 2.41 cm	0.995	0.19 cm	3.85	−2.78 cm	2.90 cm
Mean of three trials							
Sit and reach test	24.49 ± 6.58 cm	24.85 ± 6.9 cm	0.996	0.43 cm	1.73	−1.95 cm	2.67 cm
Back scratch test—PH	6.80 ± 3.42 cm	6.5 ± 3.4 cm	0.995	0.24 cm	3.63	−2.75 cm	2.15 cm
Back scratch test—NPH	4.73 ± 2.60 cm	4.5 ± 2.4 cm	0.994	0.19 cm	4.20	−2.78 cm	2.32 cm

ICC: intraclass correlation coefficient, 95% CI: 95% confidence interval, SEM: standard error of measurement, PH: preferred hand, NPH: non-preferred hand.

**Table 11:** Test–retest values (mean ± SD), as well as absolute and relative reliability indices, in boy taekwondo athletes (per age group).

Children							
	Test	Retest	ICC	SEM	SEM%	95% LOA Lower	95% LOA Upper
Single trial							
Sit and reach test	14.47 ± 7.36 cm	15.00 ± 7.00 cm	0.997	0.39 cm	2.67	−2.02 cm	3.08 cm
Back scratch test—PH	2.84 ± 2.39 cm	2.78 ± 2.1 cm	0.997	0.12 cm	4.38	−2.80 cm	2.68 cm
Back scratch test—NPH	0.95 ± 1.50 cm	0.98 ± 1.2 cm	0.998	0.06 cm	6.26	−2.91 cm	2.97 cm
Best of three trials							
Sit and reach test	14.57 ± 7.30 cm	15.01 ± 7.1 cm	0.997	0.39 cm	2.67	−2.11 cm	2.99 cm
Back scratch test—PH	2.85 ± 2.35 cm	2.80 ± 2.2 cm	0.997	0.12 cm	4.41	−2.89 cm	2.79 cm
Back scratch test—NPH	1.00 ± 1.50 cm	0.99 ± 1.15 cm	0.998	0.06 cm	5.96	−2.95 cm	2.93 cm
Mean of three trials							
Sit and reach test	14.47 ± 7.48 cm	14.65 ± 7.5 cm	0.997	0.41 cm	2.82	−2.27 cm	2.63 cm
Back scratch test—PH	2.74 ± 2.56 cm	2.68 ± 2.0 cm	0.996	0.14 cm	5.32	−2.61 cm	2.48 cm
Back scratch test—NPH	0.96 ± 1.48 cm	0.94 ± 1.3 cm	0.998	0.06 cm	6.55	−2.86 cm	2.82 cm
Adolescents							
	Test	Retest	ICC	SEM	SEM%	95% LOA Lower	95% LOA Upper
Single trial							
Sit and reach test	14.86 ± 6.58 cm	15.1 ± 6.3 cm	0.998	0.29 cm	1.92	−1.72 cm	2.2 cm
Back scratch test—PH	6.65 ± 5.60 cm	6.2 ± 5.00 cm	0.998	0.24 cm	3.69	−3.59 cm	2.69 cm
Back scratch test—NPH	2.68 ± 3.06 cm	2.7 ± 2.5 cm	0.998	0.12 cm	4.62	−2.72 cm	2.76 cm
Best of three trials							
Sit and reach test	15.20 ± 6.50 cm	15.1 ± 6.2 cm	0.998	0.28 cm	1.87	−2.75 cm	2.55 cm
Back scratch test—PH	6.70 ± 5.60 cm	6.8 ± 5.1 cm	0.998	0.24 cm	3.54	−2.64 cm	2.84 cm
Back scratch test—NPH	2.73 ± 3.04 cm	2.71 ± 2.51 cm	0.998	0.12 cm	4.56	−2.86 cm	2.82 cm
Mean of three trials							
Sit and reach test	15.00 ± 6.68 cm	14.95 ± 6.5 cm	0.998	0.29 cm	1.97	−2.31 cm	2.20 cm
Back scratch test—PH	6.55 ± 5.53 cm	6.66 ± 5.2 cm	0.998	0.24 cm	3.63	−2.26 cm	2.48 cm
Back scratch test—NPH	2.71 ± 3.02 cm	2.65 ± 2.95 cm	0.998	0.13 cm	4.98	−2.59 cm	2.47 cm

ICC: intraclass correlation coefficient, 95% CI: 95% confidence interval, SEM: standard error of measurement, PH: preferred hand, NPH: non-preferred hand.

**Table 12:** Test–retest values (mean ± SD), as well as absolute and relative reliability indices, in girl taekwondo athletes (per age group).

Children							
	Test	Retest	ICC	SEM	SEM%	95% LOA Lower	95% LOA Upper
Single trial							
Sit and reach test	19.94 ± 5.60 cm	19.7 ± 5.9 cm	0.996	0.36 cm	1.83	−2.40 cm	1.92 cm
Back scratch test—PH	3.26 ± 2.63 cm	3.1 ± 2.5 cm	0.997	0.14 cm	4.42	−2.71 cm	2.39 cm
Back scratch test—NPH	1.80 ± 2.60 cm	1.95 ± 2.00 cm	0.997	0.13 cm	6.72	−2.79 cm	3.09 cm
Best of three trials							
Sit and reach test	20.2 ± 6.73 cm	20.1 ± 6.6 cm	0.996	0.43 cm	2.09	−2.71 cm	2.51 cm
Back scratch test—PH	3.27 ± 3.0 cm	3.33 ± 3.0 cm	0.997	0.16 cm	4.98	−2.39 cm	2.51 cm
Back scratch test—NPH	1.83 ± 2.35 cm	1.96 ± 2.3 cm	0.998	0.10 cm	5.49	−2.42 cm	2.68 cm
Mean of three trials							
Sit and reach test	20.02 ± 5.6 cm	19.8 ± 5.8 cm	0.995	0.40 cm	2.03	−2.77 cm	2.33 cm
Back scratch test—PH	3.23 ± 2.94 cm	3.31 ± 2.3 cm	0.996	0.17 cm	5.07	−2.88 cm	3.04 cm
Back scratch test—NPH	1.78 ± 2.42 cm	1.8 ± 2.00 cm	0.997	0.12 cm	6.76	−3.02 cm	3.05 cm
Adolescents							
	Test	Retest	ICC	SEM	SEM%	95% LOA Lower	95% LOA Upper
Single trial							
Sit and reach test	24.05 ± 6.04 cm	24.00 ± 6.4 cm	0.998	0.28 cm	1.16	−2.40 cm	2.30 cm
Back scratch test—PH	6.53 ± 4.03 cm	6.9 ± 3.4 cm	0.996	0.23 cm	3.50	−2.18 cm	2.92 cm
Back scratch test—NPH	3.51 ± 4.04 cm	3.65 ± 3.1 cm	0.998	0.16 cm	4.46	−2.31 cm	2.59 cm
Best of three trials							
Sit and reach test	24.11 ± 6.12 cm	24.08 ± 6.2 cm	0.997	0.34 cm	1.40	−2.56 cm	2.50 cm
Back scratch test—PH	6.8 ± 4.1 cm	6.93 ± 4.23 cm	0.998	0.19 cm	2.71	−2.26 cm	2.52 cm
Back scratch test—NPH	3.53 ± 3.71 cm	3.68 ± 3.65 cm	0.998	0.16 cm	4.64	−2.3 cm	2.6 cm
Mean of three trials							
Sit and reach test	24.07 ± 6.06 cm	23.95 ± 6.1 cm	0.997	0.33 cm	1.39	−2.55 cm	2.31 cm
Back scratch test—PH	6.68 ± 4.04 cm	6.85 ± 4.1 cm	0.996	0.26 cm	3.80	−2.39 cm	2.74 cm
Back scratch test—NPH	3.46 ± 4.02 cm	3.57 ± 3.8 cm	0.998	0.17 cm	4.97	−2.52 cm	2.73 cm

ICC: intraclass correlation coefficient, 95% CI: 95% confidence interval, SEM: standard error of measurement, PH: preferred hand, NPH: non-preferred hand.

**Table 13:** Test–retest values (mean ± SD), as well as absolute and relative reliability indices, in boy non-athletes (per age group).

Children							
	Test	Retest	ICC	SEM	SEM%	95% LOA Lower	95% LOA Upper
Single trial							
Sit and reach test	9.63 ± 5.68 cm	9.5 ± 6.5 cm	0.994	0.47 cm	4.93	−2.87 cm	2.61 cm
Back scratch test—PH	2.04 ± 1.87 cm	1.95 ± 1.8 cm	0.993	0.15 cm	7.70	−3.03 cm	2.85 cm
Back scratch test—NPH	−0.2 ± 0.7 cm	−0.5 ± 0.5 cm	0.997	0.03 cm	9.39	−3.44 cm	2.84 cm
Best of three trials							
Sit and reach test	9.64 ± 5.75 cm	9.55 ± 5.74 cm	0.995	0.41 cm	4.23	−2.74 cm	2.565 cm
Back scratch test—PH	2.05 ± 2.01 cm	1.95 ± 1.98 cm	0.995	0.14 cm	7.05	−2.84 cm	2.64 cm
Back scratch test—NPH	−0.41 ± 0.7 cm	−0.5 ± 0.64 cm	0.996	0.04 cm	9.31	−2.97 cm	2.79 cm
Mean of three trials							
Sit and reach test	9.55 ± 5.61 cm	9.45 ± 5.57 cm	0.994	0.43 cm	4.56	−2.74 cm	2.55 cm
Back scratch test—PH	1.88 ± 1.88 cm	1.90 ± 1.91 cm	0.995	0.13 cm	7.09	−2.72 cm	2.77 cm
Back scratch test—NPH	−0.38 ± 0.68 cm	−0.35 ± 0.61 cm	0.997	0.04 cm	9.66	−2.75 cm	2.82 cm
Adolescents							
	Test	Retest	ICC	SEM	SEM%	95% LOA Lower	95% LOA Upper
Single trial							
Sit and reach test	9.71 ± 5.22 cm	9.9 ± 5.5 cm	0.997	0.29 cm	2.99	−2.75 cm	3.13 cm
Back scratch test—PH	2.05 ± 1.6 cm	1.99 ± 1.6 cm	0.996	0.10 cm	5.01	−3.20 cm	3.08 cm
Back scratch test—NPH	−0.17 ± 0.38 cm	−0.2 ± 0.3 cm	0.998	0.02 cm	8.22	−3.36 cm	3.30 cm
Best of three trials							
Sit and reach test	9.85 ± 5.35 cm	9.92 ± 5.41 cm	0.997	0.29 cm	2.98	−2.44 cm	2.58 cm
Back scratch test—PH	2.06 ± 1.6 cm	2 ± 1.65 cm	0.996	0.10 cm	5.06	−2.73 cm	2.61 cm
Back scratch test—NPH	−0.15 ± 0.28 cm	−0.13 ± 0.25 cm	0.998	0.01 cm	8.47	−2.70 cm	2.74 cm
Mean of three trials							
Sit and reach test	9.77 ± 5.2 cm	9.82 ± 5.4 cm	0.997	0.29 cm	2.96	−2.46 cm	2.56 cm
Back scratch test—PH	1.92 ± 1.43 cm	1.94 ± 1.62 cm	0.996	0.10 cm	5.01	−2.64 cm	2.69 cm
Back scratch test—NPH	−0.18 ± 0.36 cm	−0.15 ± 0.28 cm	0.998	0.01 cm	8.67	−2.75 cm	2.81 cm

ICC: intraclass correlation coefficient, 95% CI: 95% confidence interval, SEM: standard error of measurement, PH: preferred hand, NPH: non-preferred hand.

**Table 14:** Test–retest values (mean ± SD), as well as absolute and relative reliability indices, in girl non-athletes (per age group).

Children							
	Test	Retest	ICC	SEM	SEM%	95% LOA Lower	95% LOA Upper
Single trial							
Sit and reach test	18.30 ± 9.86 cm	18.6 ± 9.9 cm	0.995	0.70 cm	3.79	−2.64	3.24
Back scratch test—PH	1.77 ± 4.01 cm	1.85 ± 4 cm	0.998	0.18 cm	9.90	−3.06	3.22
Back scratch test—NPH	−1.61 ± 3.60 cm	−1.55 ± 3.45 cm	0.998	0.16 cm	9.98	−3.27	3.39
Best of three trials							
Sit and reach test	18.33 ± 9.99 cm	18.86 ± 9.98 cm	0.996	0.63 cm	3.40	−2.00	3.06
Back scratch test—PH	1.9 ± 4.05 cm	1.88 ± 4.00 cm	0.998	0.18 cm	9.52	−2.72	2.68
Back scratch test—NPH	−1.57 ± 3.4 cm	−1.55 ± 3.35 cm	0.998	0.15 cm	9.68	−2.82	2.86
Mean of three trials							
Sit and reach test	18.15 ± 9.80 cm	18.2 ± 9.8 cm	0.996	0.62 cm	3.41	−2.53	2.64
Back scratch test—PH	1.85 ± 4.05 cm	1.80 ± 4.02 cm	0.998	0.18 cm	9.89	−2.77	2.68
Back scratch test—NPH	−1.59 ± 3.6 cm	−1.6 ± 3.5 cm	0.998	0.16 cm	9.95	−2.81	2.79
Adolescents							
	Test	Retest	ICC	SEM	SEM%	95% LOA Lower	95% LOA Upper
Single trial							
Sit and reach test	18.50 ± 9.66 cm	19.00 ± 9.7 cm	0.996	0.61 cm	3.27	−2.05 cm	3.05 cm
Back scratch test—PH	5.34 ± 7.10 cm	5.25 ± 6.5 cm	0.997	0.37 cm	7.03	−2.93 cm	2.75 cm
Back scratch test—NPH	1.48 ± 3.01 cm	1.4 ± 2.4 cm	0.998	0.12 cm	8.40	−3.31 cm	3.15 cm
Best of three trials							
Sit and reach test	18.75 ± 9.61 cm	19.1 ± 9.5 cm	0.996	0.60 cm	3.19	−2.1 cm	2.8 cm
Back scratch test—PH	5.45 ± 7.1 cm	5.4 ± 7.02 cm	0.997	0.39 cm	7.13	−2.60 cm	2.50 cm
Back scratch test—NPH	1.5 ± 2.72 cm	1.43 ± 2.7 cm	0.998	0.12 cm	8.27	−2.77 cm	2.63 cm
Mean of three trials							
Sit and reach test	18.52 ± 9.51 cm	18.75 ± 9.6 cm	0.996	0.60 cm	3.24	−2.22 cm	2.68 cm
Back scratch test—PH	5.42 ± 7.10 cm	5.35 ± 6.05 cm	0.996	0.42 cm	7.72	−2.61 cm	2.48 cm
Back scratch test—NPH	1.46 ± 2.65 cm	1.38 ± 2.81 cm	0.998	0.12 cm	8.60	−2.71 cm	2.55 cm

ICC: intraclass correlation coefficient, 95% CI: 95% confidence interval, SEM: standard error of measurement, PH: preferred hand, NPH: non-preferred hand.

## Data Availability

Data are unavailable due to privacy or ethical restrictions.
